# Assessing patient safety culture in 15 intensive care units: a mixed-methods study

**DOI:** 10.1186/s12913-022-07665-4

**Published:** 2022-03-01

**Authors:** Mohamed Ayoub Tlili, Wiem Aouicha, Jihene Sahli, Asma Ben Cheikh, Ali Mtiraoui, Thouraya Ajmi, Chekib Zedini, Souad Chelbi, Mohamed Ben Rejeb, Manel Mallouli

**Affiliations:** 1 Faculty of Medicine of Sousse, University of Sousse, 4002 Sousse, Tunisia; 2 Department of Family and Community Medicine, LR12ES03, 4002 Sousse, Tunisia; 3 Higher School of Health Sciences and Techniques of Sousse, University of Sousse, 4054 Sousse, Tunisia; 4 Department of Prevention and Care Safety, Sahloul University Hospital, 4054 Sousse, Tunisia

**Keywords:** Patient safety culture, Quality of care, Intensive care, Patient safety

## Abstract

**Background:**

Within hospitals, intensive care units (ICUs) are particularly high-risk areas for medical errors and adverse events that could occur due to the complexity of care and the patients’ fragile medical conditions. Assessing patient safety culture (PSC) is essential to have a broad view on patient safety issues, to orientate future improvement actions and optimize quality of care and patient safety outcomes. This study aimed at assessing PSC in 15 Tunisian ICUs using mixed methods approach.

**Methods:**

A cross-sectional mixed methods approach using a sequential explanatory design was conducted from December 2019 to January 2020. The first quantitative stage was conducted in 15 ICUs belonging to the two university hospitals in the region of Sousse (Tunisia). All the 344 healthcare professionals (clinical staff) working for more than 1 month in these ICUs were contacted in order to take part in the study. In the second qualitative stage 12 participants were interviewed based on purposive sampling.

**Results:**

All of the PSC dimensions had a score of less than 50%. The developed dimension was ‘teamwork within units’ (48.8%). The less developed dimensions were ‘frequency of event reporting’ (20.8%), ‘communication openness’ (22.2%) and ‘non-punitive response to error’ (19.7%). Interviews’ thematic analysis revealed four main themes including “Hospital management/system failure”, “Teamwork and communication”, “Error management” and “Working conditions”.

**Conclusion:**

This research revealed that PSC is still in need of improvement and provided a clearer picture of the patient safety issues that require specific attention. Improving PSC through the use of quality management and error reporting systems may help to improve patient safety outcomes.

## Background

The report of the Institute of Medicine “To Err Is Human” unleashed the debates on safe care by highlighting patient safety issues such as the high prevalence of medical errors as well as their preventability [1]. Since, providing patients with safer care is gaining a growing attention and has become a major matter and challenge for healthcare systems worldwide [1].

Among the numerous healthcare settings, Intensive Care Units (ICUs) stand out in terms of patient safety. ICUs are actually considered a high-risk environment due to the medical conditions of critically ill patients and complex clinical procedures which require multiple and particular strategies to enhance quality of care in these areas [2].

Moreover, several studies reported that Adverse Events (AEs) and medication errors are more common in ICUs compared to other hospital units which could severely threaten patient safety [3–8].

In Tunisia, a study conducted by Letaief et al. [9] to explore the incidence, typology and outcomes of AEs within a teaching hospital, revealed that 41.1% of patients admitted to ICUs have contracted at least one AE, with 70% of the AEs being preventable.

To address these issues, evidence advocates that building and developing patient safety culture (PSC) ought to be a strategic priority as it plays a vital role in promoting patient safety, quality of care and patient outcomes [10]. In ICUs, a study from the United States found that having a developed PSC reduces duration of stay and mortality rates [11].

PSC assessment is a critical first step towards optimizing PSC in healthcare settings [12, 13]. It enables for the identification of key strengths and weaknesses, as well as the adoption of initiatives that promote safe care with the goal of improving patient outcomes, minimizing avoidable healthcare-related damages, and prioritizing patient safety [7].

The vast majority of PSC studies have used quantitative methods, which are advantageous since they are cost-effective, time-efficient, and enable direct comparisons between results of different contexts [13]. Despite the relevance and importance of these research, questionnaires do not provide a complete image and an understanding of why specific responses are given [2, 13]. Hence qualitative research are still needed if we intend to acquire a deeper understand of the various facets related to this topic and to explore perceptions, feelings and personal experiences that cannot be documented by administering questionnaires with pre-established responses [2, 13].

As a result, a combined assessment that includes both quantitative and qualitative surveys is required to better understand the safety culture that reigns within the healthcare setting [14]. However, there is currently a scarcity of mixed methods research on PSC whether in general or particularly in ICUs [14]. Thus, this study aimed to determine PSC in intensive care units using a mixed-methods approach.

## Materials and methods

### Study design, setting and participants

A cross-sectional mixed methods approach using a sequential explanatory design was conducted from December 2019 to January 2020. The first quantitative stage was conducted in 15 ICUs belonging to the two university hospitals in the region of Sousse (Tunisia). ICUs specialties were pediatric, emergency, cardiological, medical and surgical ICUs in Hached university hospital and pediatric, emergency, medical, surgical, transplant, neurological, severe postoperative, adult cardiovascular and thoracic surgery, pediatric cardiovascular and thoracic surgery and cardiological ICUs in Sahloul university hospital. All the 344 healthcare professionals (clinical staff) working for more than 1 month in these ICUs were contacted in order to take part in the study. In the second qualitative stage 12 participants were interviewed based on purposive sampling.

### Data collection and integration

Data collection was sequential implying that questionnaires were distributed and collected before conducting interviews.

Integration in this explanatory sequential study involved connecting the results from the initial quantitative data analysis in the first phase to help plan the follow-up qualitative data collection phase. In fact, we analyzed the quantitative data and came up with the result that all of the PSC dimensions had a very low score. Based on a need to further understand these quantitative results, we conducted a subsequent qualitative phase that is designed to explore the results in more depth and to help explain the low scores of all the PSC dimensions so that we can obtain a more accurate and complete interpretation of the research phenomena and have a clearer vision on the current level of PSC and on the aspects of PSC that need a particular attention.

#### Quantitative phase

The Hospital Survey On Patient Safety Culture (HSOPSC) questionnaire, which is validated in French, was used in this study [15]. Due to its good psychometric qualities, it is the most often used tool to assess PSC, with a Cronbach's alpha of 0.88 for the entire questionnaire and varying between 0.46 and 0.84 for the different dimensions [15].

The HSOPSC is made up of 40 items that are organized into ten dimensions. Professionals’ perceptions on patient safety grade and the number of AEs reported in the previous 12 months were also investigated in the survey. Participants’ PSC was determined using a 5-point Likert scale with agreement (from strongly disagree to strongly agree) or frequency (from never to always) scale [15, 16].

#### Qualitative phase

Two nurse unit-managers, eight full-time nurses, and two physicians were interviewed in semi-structured individual interviews. An interview guide based on open-ended questions inspired from previous studies was developed by the authors. Interviews were organized according to the availability of participants and took place in a secluded area of the ICU. Interviews were conducted in Tunisian dialect and lasted between 20 and 50 min.

The interview began with an open question that asks the interviewee to describe the level of patient safety at their unit and why they perceive it that way. Afterwards, the interview continues based on two broad questions: “What do you think is the most essential thing your unit can do to promote patient safety?” and “What do you think is preventing your unit from improving patient safety?”. If necessary, probing questions were asked to elicit more detailed responses: “Can you develop further?”, “What exactly do you mean by this?”

Interviews continued until data saturation was reached, which was observed with participant ten; yet we decided to interview two other participants to ensure that data saturation was reached. The first author transcribed the audio-recorded interviews verbatim, which was then vetted by the participants to verify that they accurately reflected their perspectives.

### Data analysis

Data analysis was performed using SPSS 20. Percentages, frequencies, means, and standard deviations are displayed in descriptive analysis. The score of each dimension is calculated based on the average positive response rate. If a dimension has a score of 75% or higher, it is termed “developed”, and if it has a score of 50% or less, it is considered “to be improved.”

For qualitative data, interviews were analyzed using thematic analysis. Researchers transcribed interviews and read them numerous times during the study’s preparation and data immersion stages to get a broad view of the whole. The researchers created categories and subcategories using open coding and grouping throughout the organizing phase. The research team proceeded to discuss and debate the analysis until a consensual agreement was established.

### Ethical considerations

The study began after the approval of the ethics committee and the authorization of the different department heads of the units. After receiving their consent to participate in the study, the participants were given an anonymous self-reported questionnaire. All the transcriptions related to interviews were anonymized. All methods were carried out in accordance with relevant guidelines and regulations.

## Results

### HSOPSC survey

A total of 284 professionals responded to the survey, with a response rate of 82.55%. Participants’ mean age was 39.09 ± 7.382 years. Participants were distributed over 15 ICUs belonging to two university hospitals and the vast majority of them (85.9%) represented paramedical staff (nurses, specialized nurses and assistant caregivers) (Table 1).

**Table 1:** Participants’ characteristics

Characteristics	Frequency (n)	Percentage (%)
***Total***	*284*	*100*
**Gender**		
Males	92	32.4
Females	67.6	67.6
**Age**		
≤ 40 years	181	63.7
≥ 41 years	103	36.3
**Professional grade**		
Physician	40	14.1
Nurse	173	60.9
Healthcare technician	60	21.1
Assistant caregiver	11	3.9
**Work experience**		
≤ 5 years	**75**	**26.4**
6–10 years	**105**	**37**
≥ 11 years	**104**	**36.6**
**Participation into risk management committees**		
Yes	108	38
No	176	62
**Training in patient safety**		
Yes	211	74.3
No	73	25.7

### Perception of patient safety quality and the frequency of reported AEs

In half of the cases (50%), professionals rated the quality of patient safety in their ICUs as acceptable. The level of safety quality was rated as “poor” and “failing” by 39.8% of professionals. In terms of reported adverse events, 92.3% of participants stated that they had not reported any in the previous 12 months (Table 2).

**Table 2:** Participants’ perceptions of patient safety and the number of adverse events reported in the previous 12 months

**Perception of patient safety quality**	**Frequency (n)**	**Percentage (%)**
Failing	26	9.2
Poor	87	30.6
Acceptable	142	50
Very Good	24	8.5
Excellent	5	1.8
*Total*	*284*	*100*
**Number of events reported**	**Frequency (n)**	**Percentage (%)**
More than 20	0	0
6–20	0	0
3–5	5	1.8
1–2	17	6
No event reported	262	92.3
*Total*	*284*	*100*

### Scores of PSC domains

With a score of less than 50%, all PSC dimensions were deemed “to be improved.” The dimension with the highest score (48.8%) was ‘teamwork within units’ and the dimensions that were the less developed were ‘frequency of event reporting’ (20.8%), ‘communication openness’ (22.2%) and ‘non-punitive response to error’ (19.7%) (Table 3).

**Table 3:** Scores and the 10 PSC dimensions in ascending order

Scores of PSC domains	Average positive response (%)
D7: Non-punitive response to error	19.7
D2: Frequency of events reported	20.8
D6: Communication openness	22.2
D8: Staffing	27.2
D10: Teamwork across units	29.3
D1: Overall perceptions of safety	34.4
D9: Management support for patient safety	34.6
D3: Supervisor/Manager expectations and actions promoting patient safety	35.3
D4: Organizational learning and continuous improvement	37.7
D5: Teamwork within units	48.8

### Qualitative perspective on PSC

Twelve ICU professionals were interviewed: 10 nurses (5 registered nurses, 3 specialized nurses and 2 unit-managers) and two physicians. Four main themes emerged from interviews’ analysis which were “Hospital management/system failure”, “Teamwork and communication”, “Error management” and “Working conditions” (Table 4).

**Table 4:** Categories and subcategories emerging from interviews

Categories	Sub-categories
Hospital management/system failure	Integrated management system Mismanagement of material resources Training/ continuous learning
Teamwork and communication	Interprofessional collaboration Communication failure Communication openness Mutual respect and role recognition
Error management	Under-reporting Fear and blame culture Absence of learning culture
Working conditions	Workload Job satisfaction

#### Theme 1: hospital management/system failure

##### Subcategory: integrated management system

Participants find that the hospital management plays a vital role in delivering safer care and that the management of the unit and the improvement of its functioning cannot be seen and approached independently of the systemic factors related to the management of the hospital : “*Management must create a whole climate that promotes patient safety and quality of care ( … ) and be the orchestra chief*” (P7, nurse).

"*Quality improvement in the ICU will never be achieved if it is not part of a whole hospital policy and strategy. ( … ) The ICU alone and its staff cannot deliver safe care. Even with optimal care in the unit, patients’ condition may worsen as a result of poor management before admission or after discharge from the ICU.*” (P2, nurse).

##### Subcategory: mismanagement of material resources

Interviewees admit that there is a shortage of the necessary means for good practice and patient safety and that there is mismanagement and misallocation of resources.

Two nurses confirmed that the way the hospital is managed prioritizes the scarcity of expenses and the “policy of austerity” than the a priori management of adverse events:

“*When you ask for disinfectants to disinfect the room after the patient has been discharged, either they give you a product that we all know is not effective, or they don’t give it to you, saying that there is no ( … ) after, we realize that the new patient admitted has contracted the same germ as the discharged patient who was in the same room. They don't know that treating an infection costs much more than the disinfectant. This is a prime example of mismanagement* » (P3, nurse).

« *we have 11 rooms so 11 patients, you ask for 11 adhesives for pressure ulcer prevention, they only give us 10, it's as if implicitly they tell you to let the other one develop bedsores.. which costs more, treatment of pressure ulcers or adhesive bandage?* » (P8, nurse).

It was noted that staff are aware that this lack of resources is the root of many errors and adverse events but the reactions are different, sometimes staff submit to the status quo and they know that what they are doing is wrong however justified by the lack of material resources:

"imagine that the last time I passed the night shift with only 3 packs of compresses (3 * 5 compresses) and I found myself in a position to work with the same compress for 2 or 3 patients and also the same gloves since there were no more ... you think that this does not affect patient safety?, of course it does, but I don’t have a choice ” (P7, nurse).

Sometimes the staff also insists on the request for equipment and refuses to provide non-compliant care:

"*I find myself each time required to go to the depot and ‘make a scene/argument’ so I can have gloves and compresses*" (P9, nurse).

This can have consequences on a personal or professional level:

"*it exhausts me, sometimes I ask myself why am I making so much effort and putting myself in situations of fights, this if you are lucky and they do not call your supervisor to question/interrogate me*" (P1, nurse).

##### Subcategory: training/continuous improvement

Participants mentioned the insufficient possibilities for continuing education and professional development:

“*unlike other institutions in the world, it is very rare that the management organizes a seminar or training sessions either for quality and safety of care or anything else*” (P4, physician).

"*The only possibility to attend a seminar is when it is organized by another organization (association for example) but already most of the time, they refuse to grant you a leave to be able to attend because of staff shortage and there is no one ‘to replace you’* ” (P1, nurse).

"*I chose to work on night shift so that when there is a seminar, training sessions, a certificate of studies etc., I can attend freely*" (P6, nurse).

“*To come back to the financial and mismanagement problem and link it to this problem, the administration refuses to grant us support, since the seminars are very expensive .. when I do practical training, it can prevent mistakes that cost more than the training or the seminar, but who understands?* “ (P11, nurse).

#### Theme 2: teamwork and communication

##### Subcategory: Interprofessional collaboration

Another point raised concerns the teamwork in the unit:

“*I feel that everyone comes here just to mark their presence to be paid.. we work here with the spirit of each one has patients to take care of and it stops there. I don’t find that there is really a team commitment to patient safety*” (P5, nurse).

“*Even if you ask for help, it’s very rare that someone will come and help you with pleasure, you still feel very uncomfortable asking for help when you need it ..they make you feel that they offering you a favor, beyond their tasks, and not because they want to help you with a sense of team*” (P12, nurse).

The problem of teamwork between the different professional categories was also raised:

“*there is no notion of interdisciplinarity, the doctors work alone, oversee the paramedics who also work alone, we do not feel that we are a solid team that works together with a single objective, to save the patient .. , even communication between them is almost absent* ” (P11, nurse).

##### Subcategory: communication failure

In terms of communication, it was reported that important information is sometimes not exchanged between professionals:

“*a lot of information, even very important ones, are not exchanged between the team members despite the fact that we meet and discuss things outside of work. I understand that maybe everyone wants to take advantage of this time of friendliness to escape from work problems, but we still have to make a balance”* (P6, nurse)*.*

This communication problem is also found between management (direction) and staff:

“*there is no communication between the administration and the staff ( … ) they only know how to stick papers on the wall or the bulletin board without explaining to us what it is..so stick on the walls as you want, without speaking to me and I, in turn, will not read*. " (P1, nurse).

##### Subcategory: communication openness

An issue related to the open communication between team members was also raised:

"*you can't even imagine what could happen if you criticize the work of other staff if they didn't do something right, you will to be treated as if you take yourself for the expert and ‘Mr who knows everything’ and who do you think you are to criticize me and all that jargon (...)* " (P2, nurse ).

##### Subcategory: mutual respect and role recognition

This problem is accentuated between the doctors and nurses who admit that there is a real problem of respect, role recognition and underestimation:

"*if it's a doctor, it’s even worse .. he mocks you saying if it's you who is going to teach him how he does his job and show him the right from wrong?* " (P2, nurse).

“*I remember once when I opened a debate on a practice with a doctor, after a few exchanges he told me ‘Don’t cross your limits, (...) you’re here only to change the bandages, I am the doctor here!* " (P9, nurse).

#### Theme 3: error management

##### Subcategory*: underreporting*

Participants revealed an under-reporting issue:

“*we don’t have the culture of reporting errors yet*” (P10, physician)

“*errors and mistakes are very rarely reported, especially if they did not affect the patient*” (P3, nurse).

##### Subcategory: fear and blame culture

According to the interviewees, this problem of under-reporting has its origins in the culture of blame and fear that reigns in the units:

"*Why would I report a problem or an error if I will be blamed for having committed it?* " (P12, nurse)

“*I remember once that after declaring infections, a report was sent to the regional health directorate*. " (P4, physician).

"*Even in the presence of a non-punishment charter, I will never put my name as a reporter .. I do not trust this charter*" (P7, nurse).

"*I don't understand why we are blamed and pointed out when we make a mistake, as if everyone does not know that there are a lot of failures in the unit and that it's them origin of the error, not me* " (P6, nurse).

##### Subcategory: absence of learning system

The absence of a whole error management system and learning system was revealed verbatim:

"*Normally the error reported would have to be followed by analysis to find out its origin and source but that never happens here, so what's the point of the reporting?* " (P10, physician).

"*Mistake is never seen as an opportunity to learn from it, it's just seen as a lack of knowledge and expertise*" (P11, nurse).

#### Theme 4: working conditions

##### Subcategory: workload

Professionals highlighted a problem of workload claiming the shortage of staff to cope with the high workload:

“*there is too much to do with a lack of human resources, you feel really exhausted at a certain point*” (P5, nurse).

"*We have a problem with human resources, during the academic year it works because there are interns and trainee students but in the summer, with the leave, it's a hassle!* " (P8, nurse).

"*The number of patients and occupied beds is very high compared to the number of staff*" (P9, nurse)

The number of hours of work was considered to be very important to ensure quality care:

"*I find that working so many hours is not normal, it is usual that when you are exhausted, you bumble work a bit* " (P7, nurse).

##### Subcategory: job satisfaction

The interviewees also reported satisfaction at work as a related factor to patient safety underlying a lack of motivation and psychological support:

“*If we endured these miserable conditions but in return you hear beautiful words of encouragement and gratitude, it might pass. But to work under such conditions without any psychological support, at a certain point you will hate the work and the unit and you become demotivated*” (P12, nurse).

Salary satisfaction was also highlighted by most of the nurses:

“*the salary we receive is for someone who provides one tenth of the effort we provide, not to mention the risk of the job*” (P2, nurse).

## Discussion

Ensuring patient safety in ICUs has received increased focus because of the rising prevalence of AEs and the potential for serious repercussions [3–5, 17]. In these high-risk areas, a well-developed PSC is seen as the key to improving patient safety and optimizing healthcare quality [18, 19]. Aware of the importance of assessing PSC, many studies have been conducted on this topic in order to develop approaches to improve patient safety in the ICU. This study was conducted in this frame, aiming at assessing PSC in 15 ICUs using a mixed approach.

Only 34.4% of participants in our study had a positive perception of safety (D1) in their units. As well, 39.8% of professionals rated the level of safety in their units as “poor” and “failing”. This reveals that there are numerous failures and patient safety issues and it is evidence for the existence of genuine hurdles that prohibit ICU staff from doing their duties correctly to provide sure and safe care.

Results also revealed that the most developed dimension was ‘teamwork within units’ (D5) with a score of 48.8%. Such a score is nevertheless inadequate, as it reflects an alarming condition given the cruciality of teamwork within ICUs [20]. In such complex environment, patient care increasingly relies on diligent efforts synchronization amongst healthcare professionals with the highest possible level of collaboration, mutual support and information sharing [20]. Despite its importance, teamwork failures in ICUs are still a challenge as they represent the root causes of many AEs [20]. A multi-center review of incident reports from 23 ICUs showed that team-related factors contributed to 32% of incidents [21]. It was demonstrated that an improved interaction and coordination between ICU professionals was related to shorter length of stay and lower rates of periventricular/intraventricular hemorrhage or periventricular leukomalacia (PIVH/PVL) [20].

Another point that was raised by interviewees concerned the problem of teamwork between the different professional grades (nurse/physician). This problem should be taken seriously since it has major repercussions on patient care. Positive perception of nurse-physician collaboration was associated with lower rates of mortality and readmission in ICUs [20].

The less developed dimension was “Non-punitive response to error” (D7). This punitive environment of blame and fear of punishment for the ICU staff may explain the under-reporting that the second dimension “frequency of events reported” show. This issue was also revealed by qualitative data where interviewed confirmed that the problem of under-reporting has its origins in the culture of blame and fear that reigns in the units.

Among the reasons explaining under reporting, interviewees also revealed the lack of feedback and learning culture and therefore the staff no longer see the importance of reporting. According to previous studies, there are several hurdles to incident reporting, including a lack of time to report, insufficient feedback, fear of punishment and blame, and reputational and patient confidence loss [22, 23].

The dimension 9 “ Management support for patient safety” showed a score of 34.6%. Actually, management has a crucial role in improving patient safety and identifying key objectives for the development of PSC in healthcare settings. The quality of care requires a combination of the commitment of healthcare professionals, on the one hand, and management, on the other hand, in terms of safety [24]. In this context, France has set up a national program for patient safety which recognizes and promotes the importance of management in the improvement of safety and quality of care [25]. According to this program, management must be involved in the establishment of a PSC by encouraging initiatives and including the whole team in this process [25]. According to the this program and also El-Jardali F et al., [22] management should make patient safety a strategic priority, however only 38.7% find that the management of the setting creates a work environment that optimizes the safety of care and only 38.4% of the participants find that the actions carried out by the management of the institution show that the safety of care is one of its top priorities. Qualitative data deepened managerial failures by revealing misconduct of material resources. Two nurses confirmed that the way in which the hospital is managed prioritizes the scarcity of expenses and the “policy of austerity” rather than the a priori prevention of adverse events. Furthermore, it was noted that interviewees are aware that this mismanagement of material resources is the root cause of many errors and adverse events.

Additionally, interviewees mentioned the insufficient possibilities for continuing education and professional development which can have negative consequences in terms of patient safety. Continuous education enhances healthcare quality and promotes the effectiveness of patient care, resulting in patient safety being maintained and improved [26]. Health practices must be systematized, organized, and based on knowledge and experience, but they must also be updated to provide high-quality care [26]. The study by Tlili et al. also supports this, demonstrating that professionals that attended patient safety training had a more developed PSC [7].

Staffing (D8) was also revealed to be a concerning dimension; it had a score of 27.2% with responses to statements highlighting staff shortage, high level of workload and emphasized therefore the deplorable working conditions within the included ICUs. A study exploring patient safety culture in ICUs in 10 hospitals also revealed that the PSC was substantially less developed when the workload was higher, which can have negative consequences for both the staff and the patient [27].

Interviewees also reported a lack of job satisfaction underlying a lack of motivation and psychological support and dissatisfaction with remuneration. A previously published study revealed also that nurses working in ICUs were less satisfied with their job than nurses working in other hospital areas and are more likely to leave their positions [28]. It further stated that ICU staff dissatisfaction is critical since it will result in a decline in the quality of care provided to the most critically vulnerable patients [28]. Particular attention must be paid to this problem of job satisfaction and to mental health in general in such a stressful environment to prevent its serious consequences for both staff and patients.

### Implications for practice

New strategies are compulsory to enhance patient safety and quality of care. Identifying strengths and weaknesses assists in putting the focus on the aspects of patient safety requiring specific attention and in orientating improvement actions. According to the findings, it is necessary to provide basic and key competencies and skills in patient safety and quality of care to ICU personnel by including PSC into health professionals’ curriculum and as part of continuing education after graduation.

Another area of improvement concerns communication and teamwork which should be addressed by trainings sessions to foster a better grasp of teamwork principles and the development of effective communication techniques and strategies.

With regards to the under-reporting issue, professionals should feel protected; an anonymous voluntary reporting system that encourages error reporting within a climate of trust and tolerance is necessary. In order for the system to succeed, staff members who report issues must trust that senior staff and management will treat them properly and not unfairly criticize them. Senior employees and management must concentrate on all of the contributing factors rather than the person. It’s critical to search for the underlying reasons of the incident, not just the “ultimate error” and to devise an action plan that tackles them. Those who report occurrences should be notified of the investigation’s findings and the actions taken.

It’s also critical to implement a human resources management strategy, which involves properly allocating workers and working hours to better manage workload.

## Conclusion

Study results showed that all PSC domains need to be improved. However, we highlighted several areas of concern need a special attention and specific strategies of improvement such as incident reporting, blame culture and workload. This research also showed the importance of completing quantitative survey by qualitative data that deepen and help understanding the current level of PSC.

Enhancing PSC of all health professionals should be a priority and strategic axis that must be focused on by putting in place consistent approaches insisting on building a blame-free environment, teamwork and incident reporting coupled by continuous learning system.

## Data Availability

On demand, from corresponding author.

## Abbreviations

ICUs: Intensive Care Units
AE(s): Adverse Event(s)
PSC: Patient Safety Culture
HSOPSC: Hospital Survey On Patient Safety Culture

## References

[1] Ibrahim MA, Osman OB, Ahmed WA, 1 Public Health Department, Faculty of Applied Medical Sciences, Albaha University, Saudi Arabia, 2 Nursing Department, Faculty of Applied Medical Sciences, Albaha University, Saudi Arabia (2019). Assessment of patient safety measures in governmental hospitals in Al-Baha, Saudi Arabia. AIMS Public Health.

[2] Souza CS, Tomaschewski-Barlem JG, Rocha LP, Barlem EL, Silva TL, Neutzling BR (2019). Cultura de segurança em unidades de terapia intensiva: perspectiva dos profissionais de saúde. Rev Gaúcha Enferm.

[3] Minuzzi AP, Salum NC, MOH L. Assessment of patient safety culture in intensive care from the health TEAM’S perspective. Texto Contexto Enfermagem. 2016;25. 10.1590/0104-07072016001610015.

[4] Garrouste-Orgeas M, Philippart F, Bruel C, Max A, Lau N, Misset B (2012). Overview of medical errors and adverse events. Ann Intensive Care.

[5] Roque KE, Tonini T, Melo ECP. Adverse events in the intensive care unit: impact on mortality and length of stay in a prospective study. Cadernos de Saúde Pública. 2016;32. 10.1590/0102-311X00081815. 10.1590/0102-311X00081815 27783755

[6] Salem M, Labib J, Mahmoud A, Shalaby S (2019). Nurses’ perceptions of patient safety culture in intensive care units: a cross-sectional study. Open Access Maced J Med Sci.

[7] Tlili MA, Aouicha W, Ben Rejeb M, Sahli J, Ben Dhiab M, Chelbi S (2020). Assessing patient safety culture in 18 Tunisian adult intensive care units and determination of its associated factors: a multi-center study. J Crit Care.

[8] Ahmed AH, Giri J, Kashyap R, Singh B, Dong Y, Kilickaya O (2015). Outcome of adverse events and medical errors in the intensive care unit: a systematic review and Meta-analysis. Am J Med Qual.

[9] Letaief M, El Mhamdi S, El-Asady R, Siddiqi S, Abdullatif A (2010). Adverse events in a Tunisian hospital: results of a retrospective cohort study. Int J Qual Health Care.

[10] Nordin A, Theander K, Wilde-Larsson B, Nordström G (2013). Health care staffs’ perception of patient safety culture in hospital settings and factors of importance for this. Open J Nurs.

[11] Huang DT, Clermont G, Kong L, Weissfeld LA, Sexton JB, Rowan KM (2010). Intensive care unit safety culture and outcomes: a US multicenter study. Int J Qual Health Care.

[12] Nascimento A (2011). Sécurité des patients et culture de sécurité: Une revue de la littérature. Ciência &amp. Saúde Coletiva.

[13] Alqattan H, Morrison Z, Cleland JA (2019). A narrative synthesis of qualitative studies conducted to assess patient safety culture in hospital settings. Sultan Qaboos Univ Med J.

[14] Listyowardojo TA, Yan X, Leyshon S, Ray-Sannerud B, Yu XY, Zheng K (2017). A safety culture assessment by mixed methods at a public maternity and infant hospital in China. JMDH.

[15] Occelli P, Quenon J-L, Kret M, Domecq S, Delaperche F, Claverie O (2013). Validation of the French version of the hospital survey on patient safety culture questionnaire. Int J Qual Health Care.

[16] Comité de coordination de l’évaluation clinique et de la qualité en Aquitaine (2015). Mesure de la culture de sécurité des soins dans les établissements de santé: Guide d’utilisation.

[17] Mello JF, Barbosa SFF. Cultura de segurança do paciente em unidade de terapia intensiva: perspectiva da equipe de enfermagem. Revista Eletrônica de Enfermagem. 2017;19. 10.5216/ree.v19.38760.

[18] Farzi S, Farzi S, Taheri S, Ehsani M, Moladoost A. Nurses’ perspectives of patient safety culture at neonatal intensive care unit. IJN. 2017. 10.22038/ijn.2017.22713.1271.

[19] Ammouri AA, Tailakh AK, Muliira JK, Geethakrishnan R, Al Kindi SN (2015). Patient safety culture among nurses. Int Nurs Rev.

[20] Dietz AS, Pronovost PJ, Mendez-Tellez PA, Wyskiel R, Marsteller JA, Thompson DA (2014). A systematic review of teamwork in the intensive care unit: what do we know about teamwork, team tasks, and improvement strategies?. J Crit Care.

[21] Pronovost PJ, Thompson DA, Holzmueller CG, Lubomski LH, Dorman T, Dickman F (2006). Toward learning from patient safety reporting systems. J Crit Care.

[22] El-Jardali F, Jaafar M, Dimassi H, Jamal D, Hamdan R (2010). The current state of patient safety culture in Lebanese hospitals: a study at baseline. Int J Qual Health Care.

[23] Rea D, Griffiths S (2016). Patient safety in primary care: incident reporting and significant event reviews in British general practice. Health Soc Care Commun.

[24] Biggs SE, Banks TD, Davey JD, Freeman JE (2013). Safety leaders’ perceptions of safety culture in a large Australasian construction organisation. Saf Sci.

[25] Haute Autorité de Santé, Ministère chargé de la santé (2013). Programme national pour la sécurité des patients 2013–2017.

[26] Fumić N, Marinović M, Brajan D. Continuous nursing education to improve the quality of health care. Acta Med Croatica 2014;68 Suppl 1:13–16. 25326985

[27] Tlili MA, Aouicha W, Sahli J, Zedini C, Ben Dhiab M, Chelbi S, et al. A baseline assessment of patient safety culture and its associated factors from the perspective of critical care nurses: results from 10 hospitals. Aust CritCare. 2020:S1036731420303040. 10.1016/j.aucc.2020.09.004. 10.1016/j.aucc.2020.09.004 33121872

[28] Tao H, Ellenbecker CH, Wang Y, Li Y (2015). Examining perception of job satisfaction and intention to leave among ICU nurses in China. Int J Nurs Sci.

